# *De Novo* Assembly of the Common Bean Transcriptome Using Short Reads for the Discovery of Drought-Responsive Genes

**DOI:** 10.1371/journal.pone.0109262

**Published:** 2014-10-02

**Authors:** Jing Wu, Lanfen Wang, Long Li, Shumin Wang

**Affiliations:** Key Laboratory of Crop Germplasm Resources and Utilization, Ministry of Agriculture; The National Key Facility for Crop Gene Resources and Genetic Improvement, Institute of Crop Science, the Chinese Academy of Agricultural Sciences, Beijing, China; University of Western Sydney, Australia

## Abstract

The common bean (*Phaseolus vulgaris* L.) is one of the most important food legumes, far ahead of other legumes. The average grain yield of the common bean worldwide is much lower than its potential yields, primarily due to drought in the field. However, the gene network that mediates plant responses to drought stress remains largely unknown in this species. The major goals of our study are to identify a large scale of genes involved in drought stress using RNA-seq. First, we assembled 270 million high-quality trimmed reads into a non-redundant set of 62,828 unigenes, representing approximately 49 Mb of unique transcriptome sequences. Of these unigenes, 26,501 (42.2%) common bean unigenes had significant similarity with unigenes/predicted proteins from other legumes or sequenced plants. All unigenes were functionally annotated within the GO, COG and KEGG pathways. The strategy for *de novo* assembly of transcriptome data generated here will be useful in other legume plant transcriptome studies. Second, we identified 10,482 SSRs and 4,099 SNPs in transcripts. The large number of genetic markers provides a resource for gene discovery and development of functional molecular markers. Finally, we found differential expression genes (DEGs) between terminal drought and optimal irrigation treatments and between the two different genotypes Long 22-0579 (drought tolerant) and Naihua (drought sensitive). DEGs were confirmed by quantitative real-time PCR assays, which indicated that these genes are functionally associated with the drought-stress response. These resources will be helpful for basic and applied research for genome analysis and crop drought resistance improvement in the common bean.

## Introduction

The common bean (*Phaseolus vulgaris* L.), a legume native to America, is now one of the most important crops worldwide and plays an important role in solving food shortages in poor areas and adjusting the diet structure in developed countries. The global bean harvest is approximately 23 million tons, with Asia being the most important producing region with 14 million hectares, representing almost half of the global output in 2012 (FAO). However, the growth and production of the common bean are usually limited by many abiotic stresses, among which drought is the most complex and devastating on a global scale. Approximately 60% of common bean production occurs in agricultural land prone to water deficit, without irrigation systems, where unexpected drought periods result in losses that may reach up to 80% of yield reduction [1], [2]. Thus, improvement of drought resistance is a major goal for common bean breeders worldwide.

Drought is an increasingly important constraint of crop productivity and stability worldwide due to climate change. It is a physiologically complex trait and involves complex cross-talk between different regulatory levels, including adjustment of metabolism and gene expression for physiological and morphological adaptation. Previous studies are mainly focused on the traits related to drought resistance in the common bean, such as flower, seed filling, number of pods and seeds, seed weight and days to maturity [3], [4]. Traits associated with drought tolerance were identified and drought-tolerant germplasm that differed in rooting depth was identified [5]. In addition, common bean germplasm that exhibits improved levels of drought tolerance has been identified [6], [7]. Physiological analysis of common bean cultivars uncovers characteristics related to terminal drought resistance [8].

Molecular markers are powerful tools to analyze the genetic control of complex traits such as diseases resistance, seed iron and drought tolerance [9], [10], [11]. A reliable genetic map was developed to analyze the inheritance of yield traits under drought and fully irrigated conditions over three years of experiments [9]. Until now, some drought tolerance quantitative trait loci (QTL) in common beans were identified, and they were mainly associated with yield components, phenology, canopy biomass dry weight, biomass partitioning indices, stem and seed total nonstructural carbohydrate content, leaf area index, and leaf chlorophyll content and rooting pattern traits instead of photosynthate remobilization traits [9], [12]–[15]. Drought-responsive genes were increasingly described in a number of plant reviews, such as P5CS, ADC, SPDS, ZMDREB2 and OSTPS1 [16]–[21]. However, there are few studies involving gene cloning and functional verification in the common bean. *P5CS2* was isolated from the common bean and analyzed for genetic diversity [22], and a root-specific bZIP transcription factor is responsive to water deficit stress in the common bean [23]. However, the mechanisms underlying gene regulation in drought response remains elusive. In other words, drought tolerance is a cumulative process with stepwise changes in gene regulation. Therefore, the drought-induced response of the transitional landraces between drought-resistant and drought-susceptible plants may provide a better understanding of drought tolerance.

In recent years, high-throughput next generation sequencing (NGS) technologies such as Roche 454, Illumina, Solexa and ABI SOLiD have made it possible to generate gene resources at the whole genome level and to advance crop genetics and breeding with relatively low cost [24], [25]. Whole genome transcriptome analysis is an economical and effective way to exploit key factors for plant responses to biotic and abiotic stress that are involved in transcriptional and metabolic activities. These technologies have been effectively used to generate large-scale differentially expressed gene data in several plant species such as *Millettia pinnata*, cassava, hyacinth Bean, and Asian seabass [26]–[29]. Despite the common bean’s growing edible and economic importance, investigation at a comprehensive transcriptome level has been lacking. Here, we report the expression profiling of the two genotypes, Long 22-0579 (drought-tolerant) and Naihua (drought-sensitive), in response to drought stress, using RNA-seq to explore the potential candidate drought-responsive genes. The data obtained will serve as an invaluable genomic reference to further our knowledge about the common bean at the molecular level, and can be applied to molecular breeding for plants with enhanced drought tolerance.

## Materials and Methods

### Plant growth and drought treatments

In this study, we included improved cultivars of two gene pools: Andean and Mesoamerican (Table S1). Common bean cultivars, Long 22-0579 (drought-tolerant genotype) and Naihua (drought-sensitive genotype), were obtained from the National Gene Bank (China, Beijing). The seedlings were grown in plastic pots (23 cm×18 cm×18 cm) under a 14/10 h photoperiod at 25°C (day) and 20°C (night) in a greenhouse at the Institute of Crop Science, the Chinese Academy of Agricultural Sciences (China, Beijing, 116°46′E, 39°92′N). The water content of each pot was measured three times a week, and the water lost was supplemented in the pots to keep equivalent levels according to treatment requirements. A completely random block design with two treatments, terminal drought and optimal irrigation was used. Twenty-five plants were used in each treatment. All pots were irrigated to field capacity until 4 weeks after seeding. For terminal drought treatment, watering was restricted to 25% of field capacity in the pot media from 5 weeks after seeding. For optimal irrigation, pots were kept to field capacity throughout the experiment.

### Drought resistance index

Field experiments were carried out following the randomized complete block design with three replications for both control and drought stress conditions at the Institute of Crop Science, the Chinese Academy of Agricultural Sciences (China, Beijing, 116°46′E, 39°92′N). Twenty-five seeds for each line were planted, with the distance of 20 cm between the plants within a plot, and 50 cm between adjacent plots. Grain production was determined harvesting plants from the central part of the rows of each cultivar, excepting two plants as a border at each end of the row. The drought resistance index (DRI) was calculated on the basis of a multiple regression of the grain yield of stressed and unstressed plants for each cultivar [30]. DRI for individual cultivars was computed as: DRI = *Y_DS_* (*Y_DS_*/*Y_WW_*)/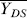 where *Y_DS_* is the grain yield obtained under unstressed per cultivar and *Y_WW_* is the yield under stressed conditions. 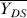 is average grain yield obtained under unstressed all cultivars.

### Sample collection and RNA preparation

The leaves were sampled when the leaves begin to wilt after the application of optimal irrigation and terminal drought treatment. Five leaves at the shoot apex were simultaneously collected from each individual plant and were frozen in liquid nitrogen and stored at −80°C prior to RNA extraction. The total RNA was isolated using TRIzol reagent (Tiangen, Beijing) following the manufacturer’s instructions. Equal amount of total RNAs from fifteen plants were pooled for a single combined sample. In total, four combined samples were collected and denoted as LOI, LTD, NOI, and NTD according to cultivar (Long 22-0579 or Naihua) and the treatments (optimal irrigation or terminal drought) of their sampling sources. For each sample, at least 20 µg of total RNA was used for Illumina Hiseq 2500 sequencing conducted at the Beijing Berry Genomics company. All sequence data have been deposited in the Short Read Archive (SRA) at the NCBI database under the project accession number SRR1523069.

### Sequences assembly

After sequencing, the raw sequence data were first purified by trimming adapter sequences and removing low-quality sequences. The resulting clean reads were assembled using the trinity software [31]. Trinity assembler was used with the inchworm k-mer method, and all of the server resources (stack size, CPU time, file size, data size, core dump size, memory usage, and virtual memory usage) were set to unlimited.

### Data analysis

SSR identification and GC content analysis were performed using in-house perl scripts. The perl script program MISA (MIcroSAtellite; http://pgrc.ipk-gatersleben.de/misa/) and SAMtools [32] was used for identification of SSRs and SNPs, respectively.

For assignments of gene descriptions, the all-unigenes were searched against the Nr database using BLASTx with an E-value cut-off of 10^−5^. Based on their annotations, the all-unigenes were assigned GO annotations using blast2GO, followed by functional classification using the WEGO software. Moreover, the putative metabolic pathways for the all-unigenes were assigned by performing BLASTx against the KEGG and COG pathway database with an E-value cut-off of 10^−5^. The RPKMs (reads per kilobase per million reads) were applied to measure the gene expression levels. The differentially expressed genes (DEGs) between the optimal irrigation and terminal drought samples were identified using ‘DEGseq’. Four separate differential expression tests implemented by DEGseq and the corresponding significance thresholds used were likelihood ratio test (LRT), Fisher’s exact test (FET) and the MA-plot-based method with random sampling model (MARS) (p-value≤0.001) and fold-change threshold on the MA-plot (FC) log_2_ normalized fold change ≥2. The GO enrichment analysis and KEGG pathway enrichment analysis for the DEGs were both performed by conducting hypergeometric tests with the whole common bean transcriptome set as the background.

### Quantitative real-time PCR analysis

qRT-PCR was conducted using the common bean actin gene (EU369188.1) as the control. The first-strand cDNAs were synthesized from 1 µg of total RNAs using the SuperScript II reverse transcriptase kit (Invitrogen). The gene-specific primers were designed using Primer Premier software (version 5.0). Real-time PCR was performed on an ABI PRISM 7300 Sequence Detection System (Applied Biosystems) using 1 µl of first-strand cDNA and SYBR Premix Ex Taq (TAKARA). All reactions were performed in triplicate. The relative expression levels for each gene were calculated using the 2^−ΔΔCT^ method with normalization to the internal control.

## Results

### Comparison of drought tolerance between Long 22-0579 and Naihua

**Figure 1:**
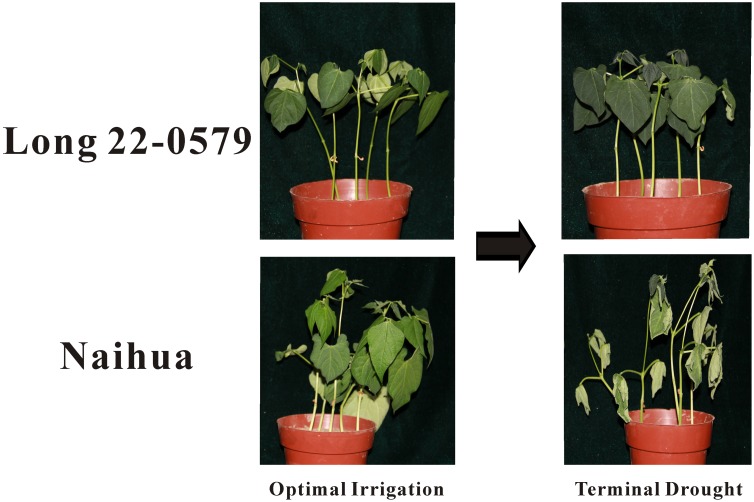
Phenotype of Long 22-0579 and Naihua genotypes after drought-stress treatment.

First, we evaluated drought resistance of one hundreds common bean cultivars by DRI value. According to the DRI values, cultivars Long 22-0579 and Naihua were the most contrasting (Table S2). Long 22-0579 in terms of yield under terminal drought was tolerant to optimal irrigation showed the highest DRI value (1.25) (Table S2). In contrast, Naihua which had the lowest seed yield under stress had lower DRI value (0.19) (Table S2). Based on screening experiments, we selected one drought-tolerant genotype (Long 22-0579) and one drought-sensitive genotype (Naihua). Further screening experiments revealed that the young seedling of the Naihua plants treated with terminal drought began to wilt after treatment. Meanwhile, the Long 22-0579 plants showed no symptoms during the course of the terminal drought treatments. These results indicate that the Long 22-0579 plants exhibit stronger drought tolerance than Naihua plants (Figure 1). Based on these results, these two genotypes (Long 22-0579 and Naihua) were chosen for RNA-seq.

### Generation and assembly of transcript reads

To obtain a general overview of the common bean transcriptome and an initial comparison between drought-resistant and drought-susceptible bean transcripts, four libraries (LOI, LTD, NOI, and NTD) were constructed for paired end (PE) sequencing. The Illumina sequencing was then performed separately for four cDNA libraries and four sub-transcriptomes with 100-bp raw reads were generated. After filtration of low-quality and adapter sequences, a total of 54 055 718, 72 287 786, 71 518 270 and 72 100 736 raw reads were generated from the Illumina GAIIx sequencer, accounting for approximately 5.4 Gb, 7.2 Gb, 7.2 Gb and 7.2 Gb of sequence data for the library cultivars LOI, LTD, NOI, and NTD, respectively. These processed paired-end reads with high quality were used for further analysis (Table S3). In total, these clean reads constitute ∼27.0 GB of sequence data. The GC contents of raw reads were from approximately 45% to approximately 47%.

Nearly 270 million clean reads from LOI, LTD, NOI, and NTD sub-transcriptomes were assembled and 139,487 transcripts were obtained. The longest transcript length was 25,393 bp and the mean transcript length was 1,487 bp. The average GC content of the common bean transcript was 39.3%. Clustering resulted in 62,828 unigenes. The longest unigene length was 14,799 bp and the mean unigenes length was 777 bp (Table 1). Among these unigenes, 34,858 (55.5%) were from 200 bp to 500 bp, 13,062 (20.8%) were longer than 500 bp, 9,648 (15.4%) were longer than 1,000 bp, and 5,260 (8.4%) were longer than 2,000 bp. Figure S1 shows that all-unigene length distributions range from 200 bases to more than 2,000 bases. The average GC content of the common bean unigenes (36%), and soybean unigenes (legume reference, 40.9%) was slightly lower than that of *Arabidopsis* (dicot reference, 42.5%) and rice (monocot reference, 55%) as reported previously [33], [34].

**Table 1:** Statistics for the unigenes of the common bean.

Length of unigenes (bp)	Number of unigenes
**200–500**	34,858 (55.5%)
**500–1000**	13,062 (20.8%)
**1000–2000**	9,618(15.4%)
**≥2000**	5,260 (8.4%)
**Total**	62,828
**Longest length**	14,799 bp
**Mean length**	777 bp
**N50**	1,321 bp
**Total length**	48,789,691 bp

### Frequency and distribution of EST-SSRs and SNPs in the common bean transcriptome

Transcriptome sequencing generated a high quantity of data in which different types of polymorphisms (e.g., EST-SSRs and SNPs) can be observed, thus providing valuable resources for the development of molecular markers. The molecular markers are important resources for marker-assisted breeding, determining functional genetic variation and map-based cloning genes. Here, we investigated two types of putative markers from common bean leaves: EST-SSRs and SNPs. Both need future validation for practical use in common bean breeding and research.

EST-SSRs are highly polymorphic, easier to develop and serve as rich resources for diversity. We identified a total of 10,482 SSR loci in transcripts of the common bean with a frequency of one SSR per 4.70 kb of sequence (Table S4). The mononucleotide SSRs represented the largest fraction (58.5%) of SSRs identified, followed by dinucleotide (20.5%) and tri-nucleotide (19.8%) SSRs. Although only a small fraction of tetra- (106), penta- (13) and hexa-nucleotide (10) SSRs were identified in common bean transcripts, the number is quite significant.

The frequencies of EST-SSRs with different numbers of tandem repeats were calculated as shown in Table S6. The most common SSRs were those with six tandem repeats (33.6%), followed by five tandem repeats (29.6%), seven tandem repeats (17.4%), eight tandem repeats (8.7%), nine tandem repeats (5.7%), ten tandem repeats (3.6%), and more than 10 tandem repeats (1.5%). The dominant repeat motif in EST-SSRs was AG/CT (32.9%), followed by AAG/CTT (16.8%), AT/TA (10.5%), AC/GT (7.5%), and AGT/ACT (7.3%) (Table S5). However, very few CG/CG (0.1%) repeats were identified in this study.

SNP markers offer the promise of higher map resolution, higher throughput, lower cost and a lower error rate. Between Long 22-0579 and Naihua, 4,099 SNPs were predicted. Approximately 82.7% of the SNPs were single-base changes of which 44.0% were transitions and 38.8% were transversions. The remaining 17.3% SNPs were indels. Of a total of 1,802 transitions, a similar proportion of bi-allelic types were detected, i.e., 896 for A/G and 906 for C/T. In 1,589 transversion mutations, four types of base substitution (T/G, G/C, A/T, A/C) were detected (Table S6).

### Gene annotation and functional classification

To identify the putative function of common bean unigenes, they were BLASTed against the NCBI non-redundant (Nr) protein sequences database. A total of 26,501 (42.2%) common bean unigenes that showed significant similarity to the proteins in Nr database were assigned Nr annotations. The majority of these annotated unigenes had the highest homology to genes from plants, and only 263 (0.99%) annotated unigenes were annotated with sequences from the non-plant sources, such as *Candida albicans*, *Actinomyces turicensis*, etc. Three-quarters of annotated unigenes retrieved annotations from *Glycine max*, reflecting the evolutionary relationship between the common bean and soybean. Moreover, there were also 1,098 annotated unigenes with matched accessions from *Phaseolus vulgaris*. Small percentages of annotated unigenes showed similarity with proteins from other legumes, such as *Cicer arietinum* (5.43%), *Medicago truncatula* (3.75%), *Lotus japonicas* (1.03%) and *Vigna* (0.46%), etc. In addition, many common bean unigenes showed homology to uncharacterized proteins annotated as unknown hypothetical and expressed proteins (Table S7).

**Figure 2:**
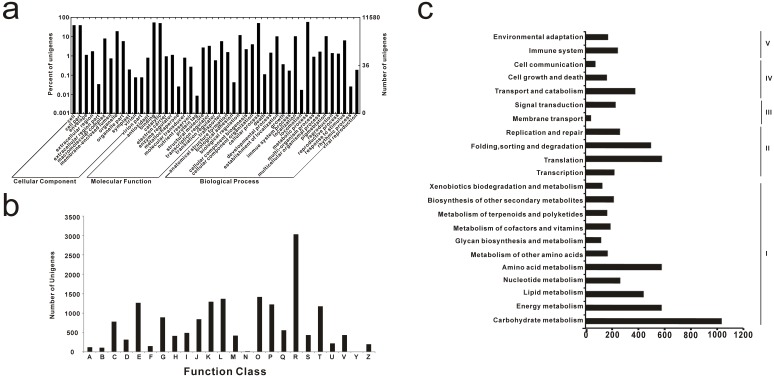
Annotation of common bean unigenes. a), GO annotation of common bean unigenes. The results are summarized in three main categories: biological process, cellular component, and molecular function. In total, 11,580 genes have been assigned 50 GO terms. In some cases, one gene has multiple terms. b), COG functional annotations of common bean unigenes. A, RNA processing and modification; B, Chromatin structure and dynamics; C, Energy production and conversion; D, Cell cycle control, cell division, chromosome partitioning; E, Amino acid transport and metabolism; F, Nucleotide transport and metabolism; G, Carbohydrate transport and metabolism; H, Coenzyme transport and metabolism; I, Lipid transport and metabolism; J, Translation, ribosomal structure and biogenesis; K, Transcription; L, Replication, recombination and repair; M, Cell wall/membrane/envelope biogenesis; N, Cell motility; O, Posttranslational modification, protein turnover, chaperones; P, Inorganic ion transport and metabolism; Q, Secondary metabolites biosynthesis, transport and catabolism; R, General function prediction only; S, Function unknown; T, Signal transduction mechanisms; U, Intracellular trafficking, secretion, and vesicular transport; V, Defense mechanisms; Y, Nuclear structure; Z, Cytoskeleton. c), Histogram presentation of KEGG classification of unigenes. The all unigenes were assigned X pathways within X clades under five major categories: I, Metabolism; II, Genetic information processing; III, Environmental information processing; IV, Cellular processes; V, Organismal systems.

GO enrichment analysis was carried out to classify the gene functions of the unigenes identified. A total of 11,580 (18.4%) unigenes were assigned and classified into 50 terms from the three main categories: biological process category, molecular function category and cellular component category. The remaining unigenes failed to be assigned GO terms, which may be attributed to the lack of information on these genes (e.g., unknown, or hypothetical and expressed proteins). Among those, 20,309, 14,166 and 14,208 unigenes were assigned at least one GO term in the biological process category, molecular function category and cellular component category, respectively. In the cellular component categories, the cell (39.65%) and cell parts (39.65%) were most abundantly represented. In contrast, rare unigenes were sorted into extracellular region parts (0.03%) or extracellular region parts (0.03%). Among the various biological processes, the two most highly represented lineages were metabolic process (58.30%) and cellular process (51.20%). The genes involved in other important biological processes, such as establishment of localization, localization, pigmentation and biological regulation, were also identified through GO annotations. Similarly, catalytic activity (50.85%) and binding (54.9%) were most represented among the various molecular functions (Figure 2a).

Using COG functional classification, 8,156 (30.8%) unigenes aligned to the COG database and were classified into 24 functional categories, among which the general function prediction was the largest group (3,035 genes, 37.2%). Followings are the groups of 1) posttranslational modification, protein turnover, chaperones (1,421 genes, 17.4%); 2) replication, recombination and repair (1,371 genes, 16.8%); 3) transcription (1,295 genes, 15.9%); 4) amino acid transport and metabolism (1,271 genes, 15.6%), 5) inorganic ion transport and metabolism (1,228 genes, 15.1%); and 6) signal transduction mechanisms (1,178 genes, 14.4%). Genes annotated as “cell motility” (21 genes, 0.3%) and “nuclear structure” (7, 0.1%) represent the smallest groups predicted by COG. (Figure 2b).

To characterize the active biological pathways in the common bean, the Kyoto Encyclopedia of Genes and Genomes (KEGG) was used to analyze the pathway annotations of unigene sequences. In total, 3,444 (13.00%) sequences were aligned with the KEGG database and were assigned to 173 KEGG pathways. These pathways belonged to 22 clades under five major KEGG categories, including ‘metabolism’, ‘genetic information processing’, ‘environmental information processing’, ‘cellular processes’, and ‘organismal systems’ (Figure 2c). Among them, ‘ribosome’(246), ‘plant hormone signal transduction’(233), ‘protein processing in endoplasmic reticulum’(182), ‘starch and sucrose metabolism’(154) and ‘purine metabolism’(149) were the top five pathways most represented by all-unigenes. These results provide an advantageous resource for investigating specific processes, functions and pathways in plant drought research.

### Different expression genes (DEGs) analysis under drought conditions

**Figure 3:**
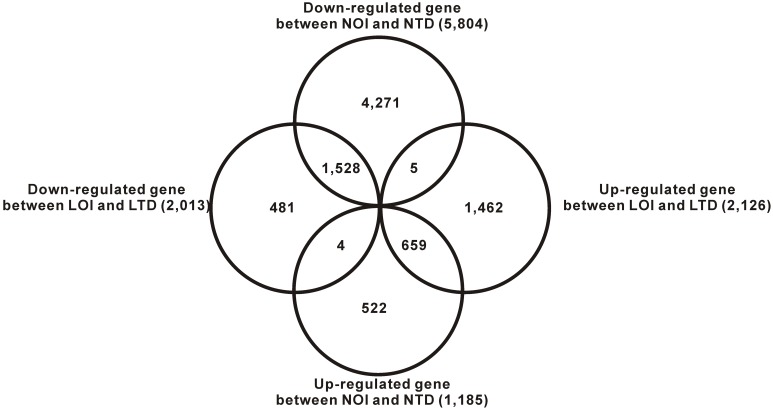
Number of DEGs in the different genotypes. The numbers of DEGs that were exclusively up- or down-regulated in one genotype are shown in each circle. The numbers of DEGs with common or opposite tendency of expression changes between different genotypes are shown in the overlapping regions. The total numbers of up- or down-regulated gene in each genotype are shown outside of the circles.

First, we performed whole transcriptome sequencing to explore whether steady-state levels of certain protein-encoding RNAs differ under drought conditions using DEGseq. We collated a list of 4,139 genes according to the DEGseq statistical tests, FET, LRT, MARS (p-value≤0.001) and FC (log2 normalized fold change ≥2), representing differences in RNA expression between samples LOI and LTD, and 6,989 genes between NOI and NTD. In this study, DEGs with higher expression levels in terminal drought-treated samples when compared with optimal irrigation-treated samples were denoted as ‘up-regulated’ versus ‘down-regulated’. Overall, there are much more DEGs in the Naihua than in the Long 22-0579. Between samples LOI and LTD, the number of down-regulated DEGs (2,013) was higher than the up-regulated DEGs (2,126). By contrast, more up-regulated DEGs (1,185) than down-regulated DEGs (5,804) were identified when comparing NOI and NTD. Only a small portion of DEGs (659 up-regulated and 1,528 down-regulated) shared common tendency of expression changes between optimal irrigation and terminal drought in Long 22-0579 or Naihua. Five DEGs were up-regulated between LOI and LTD but down-regulated between NOI and NTD, and 4 DEGs were down-regulated between LOI and LTD but up-regulated between NOI and NTD (Figure 3).

In this study, the KEGG pathway analysis helped us understand the biological function of DEGs under different treatments. Eight hundred thirty DEGs were assigned to 235 pathways, mostly associated with plant hormone signal transduction (69 DEGs), carbon metabolism (63 DEGs) and starch sucrose metabolism (60 DEGs) (Table S8). We also conducted GO enrichment analysis for DEGs with the whole transcriptome set as the background. In the biological process category, molecular function category and cellular component category, 2,899, 1,817 and 2,022 DEGs were assigned at least one GO term, respectively. The significantly overrepresented GO categories of biological processes were from single-organism metabolic pathways, oxidation-reduction pathways and carbohydrate metabolic pathways (Table S9).

After removing DEGs between treatments, the identified DEGs were analyzed using edgeR with a p-value≤0.001 and fold change ≥2 representing differences in RNA expression between different cultivars (Long 22-0579 or Naihua). DEGs with higher expression levels in Long 22-0579 when compared with Naihua were denoted as ‘up-regulated’ versus ‘down-regulated’. Overall, we collated a list of 473 genes representing differences in RNA expression between Long 22-0579 and Naihua, with more up-regulated DEGs (281) than down-regulated DEGs (192) (Table S10). With GO enrichment analysis for DEGs with the whole transcriptome set as the background, 80,151 and 3 were assigned at least one GO term in the biological processes category, molecular functions category and cellular components category, respectively. The significantly overrepresented GO terms were catalytic activity (72), oxidoreductase activity (15) and oxidation-reduction process (15) (Table S11). Twelve unigenes are related to stress response. Of the 473 DEGs, 31 were aligned with the KEGG database and were assigned to 68 KEGG pathways. The majority of the KEGG pathways included one gene, and most contained only four genes (Table S12).

### Experimental verification of DEGs

**Figure 4:**
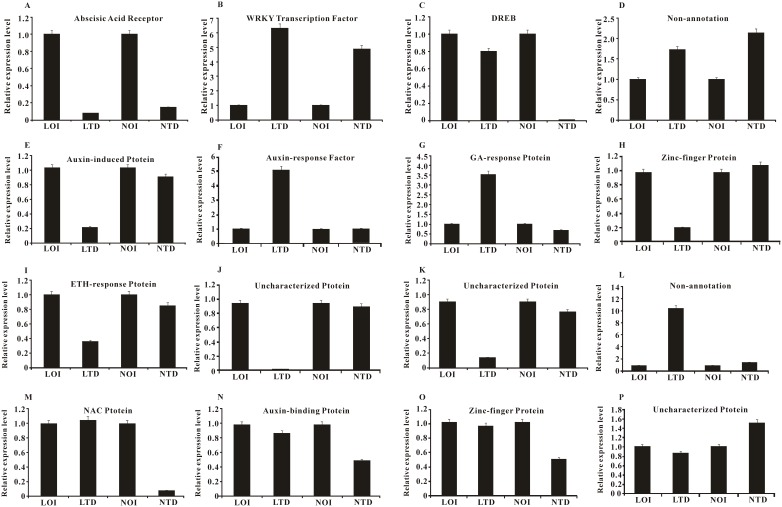
Relative expression levels of 16 DEGs. The relative gene expression levels as expressed by 2^−△△CT^ were determined separately for each treatment as the mean±S. A, *comp13117_c0_seq1*; B, *comp19490_c0_seq1*; C, *comp18282_c0_seq1*; D, *comp40404_c0_seq1*; E, *comp18253_c0_seq1*; F, *comp18888_c3_seq1*; G, *comp18293_c0_seq1*; H, *comp19585_c0_seq1*; I, *comp16965_c0_seq1*; J, *comp40354_c0_seq1*; K, *comp19389_c0_seq1*; L, *comp38170_c0_seq1*; M, *comp11891_c1_seq1*; N, *comp18632_c0_seq1*; O, *comp19229_c0_seq1*; P, *comp18242_c0_seq1*.

The 16 candidate DEGs obtained by RNA-seq analysis were further validated using RT-PCR (Figure 4). These candidates mainly included genes associated with, or involved in drought stress response in other plant species, such as genes involved in plant hormone pathways (auxin, gibberellins, and ethylene) or zinc finger proteins and genes encoding transcription factors (WRKY, NAC and DREB). In addition, there were three candidates with unknown functions and two candidates with no annotations, which represented a new resource for genes related to drought response. All these candidates fall into three classes, first, four of these genes (*comp13117_c0_seq1*, *comp19490_c0_seq1*, *comp18282_c0_seq1* and *comp40404_c0_seq1*) were shown to have differential expression between the control and treatment (Long 22-0579 or Naihua). In the case of drought, the expression level of *comp13117_c0_seq1* all up-regulated between the control and treatment (Long 22-0579 or Naihua, respectively. However, *comp19490_c0_seq1* and *comp40404_c0_seq1* genes were up-regulated after drought stress. These genes exhibited up or down-regulated under drought stress, moreover, these four genes expression pattern were similar between two different drought tolerant genotypes after drought stress. These four genes may not be responsible for the different levels of drought tolerance in Long 22-0579 and Naihua. More studies will be required before we know what the functions of these genes are and whether they relate to drought. Second, eight DEGs (*comp18253_c0_seq1*, *comp18888_c3_seq1*, *comp18293_c0_seq1*, *comp19585_c0_seq1*, *comp16965_c0_seq1*, *comp40354_c0_seq1*, *comp19389_c0_seq1*, and *comp38170_c0_seq1*) were shown to have differential expression between LOI and LTD. These genes whose expression levels changed about two to ten-fold in response to drought treatment. Thirdly, four DEGs (*comp11891_c0_seq1*, *comp18632_c0_seq1*, *comp19229_c0_seq1* and *comp18242_c0_seq1*) were shown to have differential expression between NOI and NTD. Significantly, the expression patterns of the most of the genes did not display clear correlations with the differences in drought tolerance observed in the Long 22-0579 and Naihua. Nevertheless, it is clear that the genes differ considerably between the two cultivars, suggesting that the DEGs could not totally explain the drought higher tolerance observed in Long 22-0579. Finally, we compared relative gene expression levels between drought-treated and control leaves. The expression profiles of the 16 candidates were generally in agreement with the predictions from the RNA-seq results (Table S13). These results suggested that the data obtained from the DEGs analysis were credible.

## Discussion

### Common bean transcriptome characterization

The common bean is one of the most important legume crop plants, with a high protein content and high amounts of fiber, complex carbohydrates and other dietary element proteins, making it a very important target for genomic studies, thus being used as a diploid model for soybean research [2]. For legumes such as soybean, *Medicago*, lotus, chickpea and pigeonpea, genome sequencing has been nearly completed and a vast collection of ESTs are available for functional genomic studies [35]–[40]. However, very few genomic resources, including transcript sequences, EST sequences and molecular markers are available for the common bean. We have generated more than 270 million sequence reads, approximately 27 Gb from the common bean whole plant transcriptome, representing approximately 42× sequencing depth of the common bean genome. This sequencing depth provides a greater number of repeat sequence reads for individual gene and greater accuracy and reliability of sequencing data, especially during the assembly process. Based on the final assembly results, we obtained a non-redundant set of 62,828 unigenes representing approximately 49 Mb sequences. More than half of the assembled gene sizes are less than 500 bp (55.5%), which might not represent the full-length of all genes in the database. The current minimum gene size is satisfactory for future EST probe applications. Finally, we successfully constructed the first common bean whole plant unigene database deploying normalization and Illumina Hiseq 2500 sequencing technologies.

The *de novo* transcriptome assemblies are useful to develop molecular markers for further efforts in common bean breeding or molecular studies. For this purpose, a large number of EST-SSR and SNP loci were generated in this study. In contrast to the several previous publications describing the development EST-SSRs and SNPs primers [41]–[43], this study provides the first large set of potential EST-SSRs (10,482) and SNPs (4,099) using transcriptome sequences in the common bean. The number of dinucleotide SSRs was much higher than tri-nucleotide SSRs in this study, which is in contrast to a previous study reporting the larger number of tri-nucleotide (53.8%) SSRs than dinucleotide (30.9%) SSRs in common bean ESTs [41]. In support of our study, the larger number of dinucleotide (31.3%) SSRs than tri-nucleotide (22.3%) SSRs has also been reported in common bean ESTs [44]. Among the tri-nucleotide and dinucleotide motifs, it appears that AG/CT, AAG/CTT was more prevalent than other motifs, as reported by Blair et al [41]. In this study, the proportions of transitions and transversions were 44.0 and 38.8%, respectively. The proportion of SNP classes detected here was similar to that previously reported in the common bean (43.57% transitions, 38.41% transversions, and 18.02% indels), but differed significantly from what was previously reported in the common bean: 43.57% transitions, 38.41% transversions, and 18.02% indels or 55.70% transitions, 44.30% transversions, and 15.00% indels [45], [46]. These genome-wide markers will enrich the existing common bean resources, and could especially be of great value for constructing high-density linkage maps, marker-trait association, diversity analysis, etc.

### Identification of drought-responsive genes

In this study, RNA-Seq DEG technology was used to identify DEGs of the common bean under optimal irrigation and terminal drought conditions. This is the first report to identify common bean drought-responsive regulatory proteins using drought-tolerant and drought-sensitive genotypes, despite the reports that the identification of DEGs related to drought stress in *Arabidopsis*, rice, and other legume plants [29], [47]–[50]. The identification of candidate genes would aid the research of common bean drought-tolerant molecular mechanisms and, more broadly, drought stress responses in legume plants. In this paper, we detected DEGs by different method. Nevertheless, it could help us to found more DEGs about drought. This strategy may be detected two type DEGs, which were identified to be differentially expressed following drought treatment in drought tolerant or sensitive genotype, and between different drought tolerant genotypes.

Drought tolerance is a complex trait that involves multiple complex molecular mechanisms to avoid or tolerate periods of water deficit. Based on the comparison of gene expression levels, we identified 9,298 drought-responsive candidate genes, approximately 14.8% of the total all-unigenes, which can be divided into two types: DEGs between treatment and control and DEGs between different genotypes. Among them, there were many homologs of drought-inducible genes that were identified previously in other plant species, such as *Arabidopsis* and soybean. According to the GO and KEGG annotations, many DEGs are involved in important drought-related metabolic processes, such as cell metabolic processes and cell wall and carbohydrate biosynthetic processes (comp34280_c0_seq1, comp3320_c0_seq1 and comp34280_c0_seq1, etc.). Cell wall genes encode proteins that function to enhancing mechanical resistance of drought-exposed cells [51]. Carbohydrate metabolism-related genes responded greatly to drought stress and were reported to enhance the capacity of the leaf lamina of *Arabidopsis thaliana* to endure a transient water deficit [52]. In this study, we also detected several proline-related genes: comp25511_c0_seq1, comp37160_c1_seq1, comp18574_c1_seq1, etc. Proline is an osmo-protectant critical for adaptation to drought [53]. Many proline-related genes have been cloned and verified to improve drought resistance of plants [54], [55]. Some transcription factor (TF) were enriched in MYB, WRKY, C2H2, NAC, and DREB family of DEGs, such as comp32316_c1_seq1, comp40771_c0_seq1, comp7618_c0_seq1, comp27508_c1_seq1 and comp34578_c1_seq1. Previous studies have shown that TF families, such as MYB, DREB, NAC, bZIP and WRKY, are directly or indirectly involved in the regulation of plant response to drought stress [56]–[58]. Recently, it has been shown that GmDREB2, GmERF3, GmWRKY13, GmWRKY21, GmWRKY54, and GsZFP1 confer the tolerance to drought in transgenic plants [59]–[62] differentially. In addition, several plant hormone-related genes, such as gibberellin biosynthesis genes (comp34755_c0_seq1), auxin related protein (comp17800_c0_seq1), ABA related genes (comp24144_c1_seq1), and ethylene related genes (comp40878_c0_seq1) were identified to be significantly and differentially between drought treatment and control. Overexpression of dehydration-responsive element-binding proteins (SlDREB) in tomato suppresses GA biosynthesis and promotes drought resistance [63]. Similarly, tomato plants overexpressing *Arabidopsis* gibberellin methyl transferase 1 (*AtGAMT1*) also exhibited increased tolerance to drought [64]. Other plant hormone-related genes were reported to play an important role under terminal drought condition [20], [65]–[66]. In addition to these genes, many other important genes were up- or down-regulated under drought, such as signaling and cell communication-related genes, including Ca^2+^-binding (comp18500_c0_seq1) and GTP binding protein (comp16864_c1_seq1), kinase and protein phosphatase (comp5659_c0_seq1), etc. These kinase and phosphatase transcripts have been suggested to impact the drought process in plants [67]–[69]. Some of the differentially expressed genes during the terminal drought process could not be annotated; therefore, their functions remain to be investigated in future study.

In conclusion, we have obtained a comprehensive transcriptome of the common bean using the Illumina sequencing technology. This study will contribute a significant, non-redundant set of 62,828 unigenes in the common bean. The detailed analyses have identified a large number of EST-SSRs and SNPs. Our work will be a great help to the development of genomic resources for the common bean and will accelerate functional genomics studies and breeding programs. In addition, the candidate drought-responsive genes identified in the common bean will be a new resource for molecular breeding in legumes or other crops. Moreover, our work has demonstrated the high reliability of the sequencing-based approach for identifying stress-responsive genes in legumes.

## Supporting Information

Figure S1 **Length distributions of unigenes.**
(JPG)

Table S1 **Classification and agronomic traits of two common bean cultivars.**
(DOC)

Table S2 **Drought resistance index of each cultivar.**
(DOC)

Table S3 **Summary of sequencing outputs.**
(DOC)

Table S4 **Statistics of SSRs identified in common bean transcripts.**
(DOC)

Table S5 **Frequency of di- and tri-nucleotide EST-SSR repeat motifs in the common bean.**
(DOC)

Table S6 **Statistics for SNPs between Long 22–0579 and Naihua.**
(DOC)

Table S7 **Annotation sources for the unigenes of the common bean.**
(DOC)

Table S8 **KEGG annotation of DEGs.**
(XLS)

Table S9 **GO annotation of DEGs.**
(XLS)

Table S10 **DEGs between Long 22–0579 and Naihua.**
(XLS)

Table S11 **GO annotation of DEGS.**
(XLS)

Table S12 **KEGG classification of DEGs.**
(XLS)

Table S13 **qRT-PCR verification of 16 DEGs in the drought-treated leaves compared to the control.**
(DOC)
