# Correction: *De Novo* Assembly of the Common Bean Transcriptome Using Short Reads for the Discovery of Drought-Responsive Genes

**DOI:** 10.1371/journal.pone.0119369

**Published:** 2015-03-26

**Authors:** The PLOS ONE Staff

There are errors in the Data Availability section. The correct data availability information is as follows: The authors confirm that all data underlying the findings are fully available without restriction. All sequence data are in the Short Read Archive (SRA) at the NCBI database under accession numbers: bean LTD SAMN03223377, bean NOI SAMN03223381, bean NTD SAMN03223380, and bean LOI SAMN03223378.

## References

[1] Wu J, Wang L, Li L, Wang S (2014) *De Novo* Assembly of the Common Bean Transcriptome Using Short Reads for the Discovery of Drought-Responsive Genes. PLoS ONE 9(10): e109262 doi:10.1371/journal.pone.0109262 25275443 10.1371/journal.pone.0109262 PMC4183588

