# Supplementary figures and images for: De Novo Assembly of the Common Bean Transcriptome Using Short Reads for the Discovery of Drought-Responsive Genes
Source: PLoS One. 2014 Oct 2;9(10):e109262. doi: 10.1371/journal.pone.0109262 (PMC4183588; doi:10.1371/journal.pone.0109262)

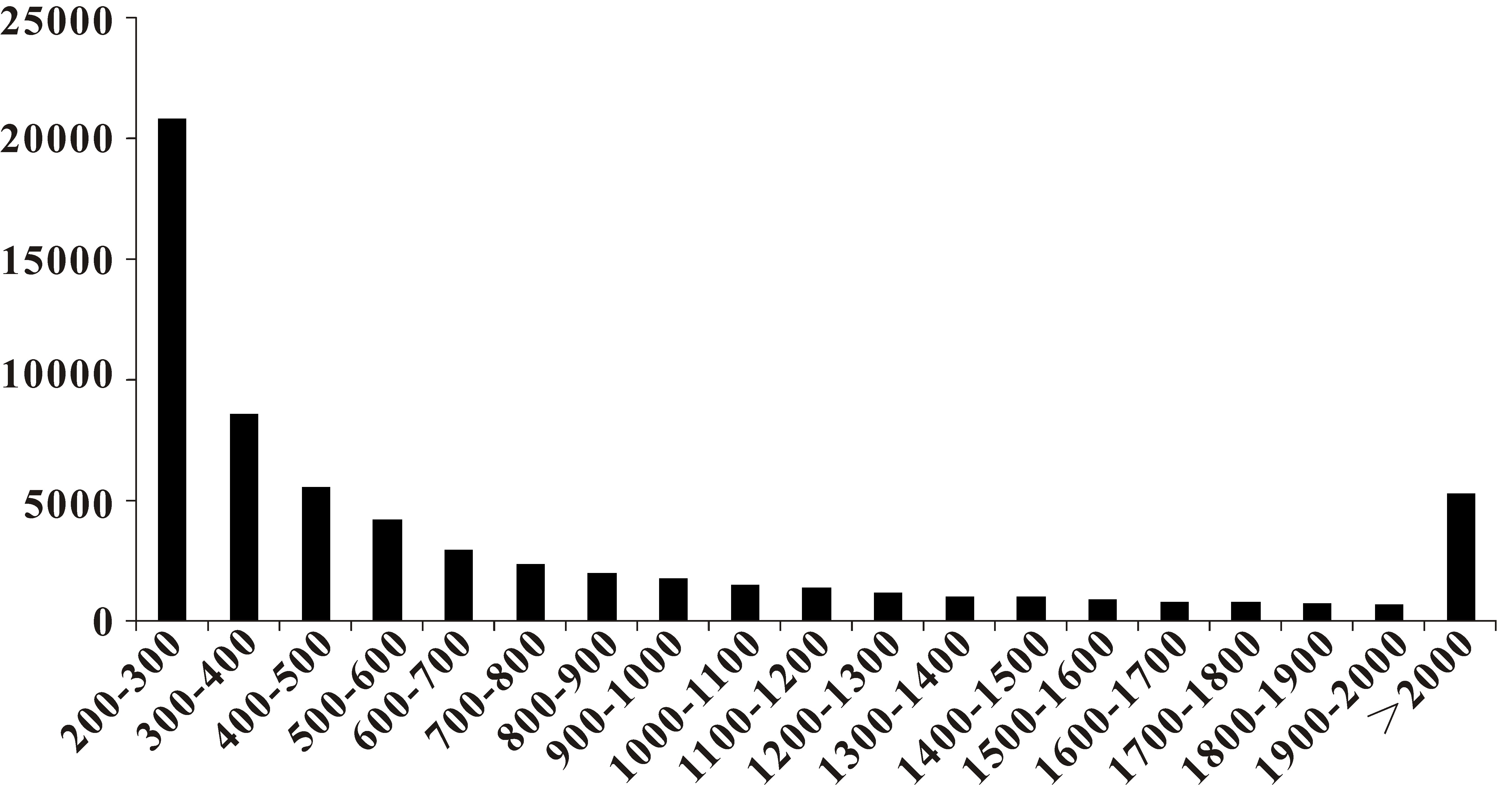

Supplement: Figure S1 — Length distributions of unigenes. (JPG) [file pone.0109262.s001.jpg]
