# Supplementary material for: De Novo Assembly of the Common Bean Transcriptome Using Short Reads for the Discovery of Drought-Responsive Genes
Source: PLoS One. 2014 Oct 2;9(10):e109262. doi: 10.1371/journal.pone.0109262 (PMC4183588; doi:10.1371/journal.pone.0109262)
Supplement: Table S1 — Classification and agronomic traits of two common bean cultivars. (DOC) [file pone.0109262.s002.doc]

**Table S1** Classification and agronomic traits of two common bean cultivars

| **Cultivar** | **Gene pool** | **Growth habit** | **Drought response** |
| --- | --- | --- | --- |
| **Long 22-0579** | Mesoamerican | Erect bush | Resistant |
| **Naihua** | Andean | Climbing | Susceptible |
