# Supplementary material for: De Novo Assembly of the Common Bean Transcriptome Using Short Reads for the Discovery of Drought-Responsive Genes
Source: PLoS One. 2014 Oct 2;9(10):e109262. doi: 10.1371/journal.pone.0109262 (PMC4183588; doi:10.1371/journal.pone.0109262)
Supplement: Table S2 — Drought resistance index of each cultivar. (DOC) [file pone.0109262.s003.doc]

**Table S2** Drought resistance index of each cultivar

| **Cultivars** | **DRI** | **Cultivars** | **DRI** | **Cultivars** | **DRI** | **Cultivars** | **DRI** |
| --- | --- | --- | --- | --- | --- | --- | --- |
| DOR-483 | 1.45 | Baiyaozidou | 0.96 | Changfandou | 0.66 | Bayuezhadouzi | 0.40 |
| Sulidou | 1.32 | Pinyun 2 | 0.96 | Sijidou | 0.65 | Huangkesijidou | 0.39 |
| Hudiedou | 1.30 | Fandou | 0.95 | Huafandou | 0.63 | Huasulidou | 0.37 |
| Sijidou | 1.28 | Suduopu | 0.93 | Dabaidou | 0.63 | Caidou | 0.37 |
| **Long22-0579** | **1.25** | Ribendou | 0.91 | Xiaojingdou | 0.63 | Naihuayundou | 0.37 |
| Baifandou | 1.24 | Hongzhuangzhuangdou | 0.90 | Hongyundou | 0.62 | Baimaodou | 0.37 |
| Sulidou | 1.23 | Heiyundou | 0.89 | Jiyaozidou | 0.60 | Huanghongdou | 0.35 |
| Wuyuexian | 1.21 | Sulidou | 0.88 | Heijindou | 0.60 | Yaozidou | 0.35 |
| Jitiandou | 1.21 | Hongdou | 0.87 | Baomidou | 0.59 | 260217 | 0.35 |
| Caidou | 1.20 | Baicaidou | 0.85 | Jiyaozidou | 0.58 | Fashiwuyacai | 0.32 |
| Heihongdou | 1.20 | Sijidou | 0.85 | Xingfuhongdou | 0.57 | SEQ1006 | 0.32 |
| 260219 | 1.20 | Naihuayundou | 0.84 | Baifandou | 0.56 | A0907 | 0.31 |
| Chenghuangjindou | 1.18 | Jinhongdou | 0.84 | FOI 10 | 0.53 | Zibaihuadou | 0.30 |
| Pinyun 1 | 1.18 | Caidou | 0.80 | BAT 58 | 0.53 | Hongfandou | 0.29 |
| FOI 11 | 1.18 | Chunchundou | 0.77 | BRB-130 | 0.52 | Naihuayundou | 0.28 |
| Xiaobaidou | 1.12 | Heicaidou | 0.74 | Habayaozidou | 0.52 | Jiaquedou | 0.28 |
| Fandou | 1.11 | DOR482 | 0.74 | Naihuayundou | 0.51 | Tiejiaziyundou | 0.24 |
| Baicaidou | 1.10 | Zimeidou | 0.72 | Zihuafandou | 0.51 | 64-92 | 0.21 |
| Dahongyaozidou | 1.06 | Tuzitui | 0.71 | Longyundou 5 | 0.50 | **Naihua** | **0.19** |
| Heiyundou | 1.06 | Xiaohuaqi | 0.70 | Xiaojinhuang 4 | 0.50 | Baiyundou | 0.18 |
| Liangshudou | 1.05 | 260200 | 0.68 | Fandou | 0.47 | Fandou | 0.17 |
| Liushitianhuanjia | 1.05 | NR | 0.68 | Heiyundou | 0.44 | Honghuayundou | 0.17 |
| Zhancaidou | 1.02 | Baijindelidou | 0.67 | Baimaodou | 0.42 | Taohuayoubailian | 0.17 |
| Fandou | 1.02 | Baiyaozidou | 0.67 | Modou | 0.41 | Huangyundou | 0.16 |
| Fandou | 1.01 | 260218 | 0.66 | Naihuayundou | 0.41 | Xiaojinhuangdou | 0.14 |
