# Supplementary material for: De Novo Assembly of the Common Bean Transcriptome Using Short Reads for the Discovery of Drought-Responsive Genes
Source: PLoS One. 2014 Oct 2;9(10):e109262. doi: 10.1371/journal.pone.0109262 (PMC4183588; doi:10.1371/journal.pone.0109262)
Supplement: Table S3 — Summary of sequencing outputs. (DOC) [file pone.0109262.s004.doc]

**Table S3** Summary of sequencing outputs

| **Sample** | **Read number** | **Total nucleotides (nt)** | **GC percentage** |
| --- | --- | --- | --- |
| **LOI** | 54,055,718 | 5,405,571,800 | 47% |
| **LTD** | 72,287,786 | 7,228,778,600 | 45% |
| **NOI** | 71,518,270 | 7,151,827,000 | 45% |
| **NTD** | 72,100,736 | 7,210,073,600 | 47% |
