# Supplementary material for: De Novo Assembly of the Common Bean Transcriptome Using Short Reads for the Discovery of Drought-Responsive Genes
Source: PLoS One. 2014 Oct 2;9(10):e109262. doi: 10.1371/journal.pone.0109262 (PMC4183588; doi:10.1371/journal.pone.0109262)
Supplement: Table S4 — Statistics of SSRs identified in common bean transcripts. (DOC) [file pone.0109262.s005.doc]

**Table S4** Statistics of SSRs identified in common bean transcripts

| **SSR mining** |  |
| --- | --- |
| Total number of sequences examined | 62,828 |
| Total size of examined sequences (bp) | 48,789,691 |
| Total number of identified SSRs | 10,482 |
| Number of SSR containing sequences | 8,836 |
| Number of sequences containing more than one SSR | 1,350 |
| Number of SSRs present in compound formation | 445 |
| Frequency of SSRs | One per 4.7Kb |
| **Distribution of SSRs in different repeat types** |  |
| Mono-nucleotide | 6,134(58.5%) |
| Di-nucleotide | 2,144(20.5%) |
| Tri-nucleotide | 2,075(19.8%) |
| Tetra-nucleotide | 106(1.0%) |
| Penta-nucleotide | 13(0.1%) |
| Hexa-nucleotide | 10(0.1%) |
