# Supplementary material for: De Novo Assembly of the Common Bean Transcriptome Using Short Reads for the Discovery of Drought-Responsive Genes
Source: PLoS One. 2014 Oct 2;9(10):e109262. doi: 10.1371/journal.pone.0109262 (PMC4183588; doi:10.1371/journal.pone.0109262)
Supplement: Table S5 — Frequency of di- and tri-nucleotide EST-SSR repeat motifs in the common bean. (DOC) [file pone.0109262.s006.doc]

**Table S5** Frequency of di- and tri-nucleotide EST-SSR repeat motifs in common bean

| **Repeat motif** | **Repeat number** | | | | | | | **Total** | **(%)** |
| --- | --- | --- | --- | --- | --- | --- | --- | --- | --- |
|  | **5** | **6** | **7** | **8** | **9** | **10** | **﹥10** |  |  |
| **AC/GT** | - | 171 | 82 | 37 | 14 | 7 | 4 | 315 | 7.5 |
| **AG/CT** | - | 511 | 294 | 240 | 180 | 121 | 40 | 1386 | 32.9 |
| **AT/TA** | - | 179 | 101 | 72 | 47 | 23 | 19 | 441 | 10.5 |
| **CG/GC** | - | 2 | - | - | - | - | - | 2 | 0.1 |
| **AAC/GTT** | 126 | 28 | 5 | - | - | - | 1 | 160 | 3.8 |
| **AAG/CTT** | 355 | 229 | 121 | 4 | - | - | - | 709 | 16.8 |
| **AAT/ATT** | 126 | 64 | 30 | 3 | - | - | - | 223 | 5.3 |
| **ACC/GGT** | 137 | 54 | 34 | 2 | - | - | - | 227 | 5.4 |
| **ACG/CGT** | 22 | 6 | 3 | - | - | - | - | 31 | 0.7 |
| **ACT/ATG** | 19 | 9 | 2 | - | - | - | - | 30 | 0.7 |
| **AGC/CGT** | 92 | 26 | 11 | - | - | - | - | 129 | 3.1 |
| **AGG/CCT** | 116 | 42 | 11 | 3 | - | - | - | 173 | 4.1 |
| **AGT/ACT** | 187 | 83 | 33 | 3 | - | - | - | 306 | 7.3 |
| **CCG/CGG** | 68 | 13 | 5 | 1 | - | - | - | 87 | 2.1 |
| **Total** | 1,248 | 1,417 | 732 | 365 | 241 | 151 | 64 | 4,219 |  |
| **%** | 29.6 | 33.6 | 17.4 | 8.7 | 5.7 | 3.6 | 1.5 |  |  |
