# Supplementary material for: De Novo Assembly of the Common Bean Transcriptome Using Short Reads for the Discovery of Drought-Responsive Genes
Source: PLoS One. 2014 Oct 2;9(10):e109262. doi: 10.1371/journal.pone.0109262 (PMC4183588; doi:10.1371/journal.pone.0109262)
Supplement: Table S6 — Statistics for SNPs between Long 22–0579 and Naihua. (DOC) [file pone.0109262.s007.doc]

**Table S6** Statistics for SNPs between Long 22-0579 and Naihua

|  | **Number of SNP** |
| --- | --- |
| **Transition** |  |
| **A/G** | 896 |
| **C/T** | 906 |
| **Total** | 1802 |
| **Transversion** |  |
| **T/G** | 333 |
| **G/C** | 344 |
| **A/T** | 540 |
| **A/C** | 372 |
| **Total** | 1589 |
| **Total** | 3391 |
