# Supplementary material for: De Novo Assembly of the Common Bean Transcriptome Using Short Reads for the Discovery of Drought-Responsive Genes
Source: PLoS One. 2014 Oct 2;9(10):e109262. doi: 10.1371/journal.pone.0109262 (PMC4183588; doi:10.1371/journal.pone.0109262)
Supplement: Table S7 — Annotation sources for the unigenes of the common bean. (DOC) [file pone.0109262.s008.doc]

**Table S7** Annotation sources for the unigenes of the common bean

| **Sources** | **Number and percent of annotated unigenes** |
| --- | --- |
| **The species with the most abundant matched unigenes** | |
| *Glycine max* | 19,983(75.40%) |
| *Cicer arietinum* | 1,439(5.43%) |
| *Phaseolus vulgaris* | 1,098(4.14%) |
| *Medicago truncatula* | 993(3.75%) |
| *Vitis vinifera* | 750(2.83%) |
| *Lotus japonicus* | 272(1.03%) |
| *Theobroma cacao* | 134(0.51%) |
| *Populus trichocarpa* | 133(0.50%) |
| *Prunus persica* | 128(0.48%) |
| *Arabidopsis thaliana* | 96(0.36%) |
| **The legume with the most abundant matched unigenes** | |
| *Glycine* | 19,992(75.44%) |
| *Cicer* | 1,439(5.43%) |
| *Phaseolus* | 1,132(4.27%) |
| *Medicago* | 999(3.77%) |
| *Lotus* | 272(1.03%) |
| *Vigna* | 121(0.46%) |
| Plants | 26,238(99.01%) |
| Non-plants | 263(0.99%) |
| Total | 26,501 |
