# Supplementary material for: De Novo Assembly of the Common Bean Transcriptome Using Short Reads for the Discovery of Drought-Responsive Genes
Source: PLoS One. 2014 Oct 2;9(10):e109262. doi: 10.1371/journal.pone.0109262 (PMC4183588; doi:10.1371/journal.pone.0109262)
Supplement: Table S13 — qRT-PCR verification of 16 DEGs in the drought-treated leaves compared to the control. (DOC) [file pone.0109262.s014.doc]

Table S 11 qRT-PCR verification of 16 DEGs in the drought-treated leaves compared to control

|  | **Gene ID** | **Gene description** | **RNA-Seq log2(FC)** | **Up /down** | **Primer Sequence (5'→3')** | |
| --- | --- | --- | --- | --- | --- | --- |
| **Fig.7A** | comp13117_c0_seq1 | abscisic acid receptor PYR1-like | LOI/LTD -3.24 | Down | F | TTGTTGGGAAGGTTGTTG |
|  |  |  | NOI/NTD -4.66 | Down | R | TTGATTGTGGGAGGGAAA |
| **Fig.7B** | comp19490_c0_seq1 | probable WRKY transcription factor 23-like | LOI/LTD 2.48 | Up | F | GCCATAGGGTCTTCGTCC |
|  |  |  | NOI/NTD 2.75 | Up | R | GAGTTCGGAGTCGCAGGT |
| **Fig.7C** | comp18282_c0_seq1 | dehydration-responsive element-binding protein 3-like | LOI/LTD -2.15 | Down | F | CGGAAGACTTGACTGAGAT |
|  |  |  | NOI/NTD -2.53 | Down | R | ATTTATACAACACGAGGTGA |
| **Fig.7D** | comp40404_c0_seq1 | Non-annotation | LOI/LTD 7.15 | Up | F | GGGGCGAAGAAGAACATA |
|  |  |  | NOI/NTD 7.99 | Up | R | CAACTTAGGGCAACACCA |
| **Fig.7E** | comp18253_c0_seq1 | Auxin-induced Protein | LOI/LTD -2.25 | Down | F | TGGGCTCCTTCTTTCTGG |
|  |  |  | NOI/NTD -1.25 | - | R | CTTCTTTGCCGTTGCTCT |
| **Fig.7F** | comp18888_c3_seq1 | Auxin-response Protein | LOI/LTD 2.12 | Up | F | AGCCTACATGCCCTGATC |
|  |  |  | NOI/NTD -0.88 | - | R | CTTAAAGCGCCATTCATA |
| **Fig.7G** | comp18293_c0_seq1 | GA-response Protein | LOI/LTD 2.92 | Up | F | GTTCCCAGTAGTAGGTTGTC |
|  |  |  | NOI/NTD -0.58 | - | R | CCTAAGGGCATGATAGATg |
| **Fig.7H** | comp19585_c0_seq1 | Zinc Finger Protein | LOI/LTD -2.25 | Down | F | GAGGGCATAATTGGGAGT |
|  |  |  | NOI/NTD 1.23 | - | R | GCATCATAAGGCAGCATC |
| **Fig.7I** | comp16965_c0_seq1 | ETH-response Protein | LOI/LTD -2.72 | Down | F | AGGGAATCACTCAAAGACA |
|  |  |  | NOI/NTD -1.68 | - | R | AAATCCTCCAACACCAAC |
| **Fig.7J** | comp40354_c0_seq1 | Uncharacterized Protein | LOI/LTD -10.70 | Down | F | GGTGCCAATGAACTTCTA |
|  |  |  | NOI/NTD 0.31 | - | R | CCTCTTGTGAGCTGCTAt |
| **Fig.7K** | comp19389_c0_seq1 | Uncharacterized Protein | LOI/LTD -2.49 | Down | F | GCTTTGTCCACTGGGTTG |
|  |  |  | NOI/NTD 0.25 | - | R | GGGTAGGCTGCTTCACTT |
| **Fig.7L** | comp38170_c1_seq1 | Non-annotation | LOI/LTD 4.39 | Up | F  R | TCCACCTTTCACGAGTTA  TCAGCAATGTTAGCCACT |
|  |  |  | NOI/NTD -1.28 | - |  |  |
| **Continued** | | | | | | |
| **Fig.7M** | comp11891_c0_seq1 | NAC Protein | NOI/NTD -3.26 | Down | F | TGGCAATAATGAAGAGGA |
|  |  |  | LOI/LTD -1.29 | - | R | AGGAATGAATGTAGCACC |
| **Fig.7N** | comp18632_c0_seq1 | Auxin-binding Protein | NOI/NTD -8.53 | Down | F | GATTCTCATTATCTGCCTCG |
|  |  |  | LOI/LTD -1.59 | - | R | ACCTGCTGCTAAGTCCAA |
| **Fig.7O** | comp19229_c0_seq1 | Zinc Finger Protein | NOI/NTD -3.41 | Down | F | ACATTGGCAGATAGCAGG |
|  |  |  | LOI/LTD -0.88 | - | R | TAGAACGCACCAAAGGAA |
| **Fig.7P** | comp18242_c0_seq1 | Uncharacterized Protein | NOI/NTD 3.41 | Up | F | TAGTACAACAATTCACAGGGTG |
|  |  |  | LOI/LTD 1.41 | - | R | AACAACTGCTGCCGAGGT |
